# Peer Review of “Influence of Mass Media on Italian Web Users During the COVID-19 Pandemic: Infodemiological Analysis”

**DOI:** 10.2196/34136

**Published:** 2021-10-18

**Keywords:** COVID-19, Google Trends, infodemiology, infoveillance, infodemic, media coverage, mass media influence, mass media, social media

*This is a peer-review report submitted for the paper “Influence of Mass Media on Italian Web Users During the COVID-19 Pandemic: Infodemiological Analysis.”*

## Round 1 Review

### General Comments

This manuscript [1] explores the very interesting topic of the impact of the COVID-19 infodemic, using Google Trends data. However, there are several points that would need to be addressed for this manuscript to become a contribution to the international literature.

### Specific Comments

#### Major Comments

1. Introduction:

The Introduction is structured like one large paragraph. It should be split. Moreover, the Introduction should be enhanced to include a concise introduction of infodemiology and previous mentions of the term *infodemic*, as well as how the term developed, what other examples in recent history we have, etc. As it is, the Introduction is somewhat poor and not very informative of the wider topic (although it has adequately addressed the Italian infodemic issue).

2. Methods:

The Google Trends methodology for data collection is also very poor and not detailed. This part should be rewritten. The *Statistical Analysis* section consists of very standard tests/correlations, and there is no need to have mathematical expressions/explanations for percentage increase, as an example.

3. Results:

The *Results* section is informative and elaborate, but at points it is too elaborate and also needs to be rewritten in order to not consist of redundant information (the additional information could be moved to an appendix).

4. Discussion:

The *Discussion* section is also not reader friendly and should be restructured, while several parts should be rewritten in order to keep the interest of the reader.

#### Minor Comments

5. Although marginally exceeding the Journal’s 450-word requirement [2], I would suggest that the Abstract be even smaller in size.

6. Try to avoid bullet points, unless absolutely necessary.

7. Cite URLs as standard references at the reference list (see Journal requirements [2]).
